# Comparison of Deep Inspiration Breath Hold Versus Free Breathing in Radiotherapy for Left Sided Breast Cancer

**DOI:** 10.3389/fonc.2022.845037

**Published:** 2022-04-21

**Authors:** Yongkai Lu, Di Yang, Xiaowei Zhang, Yonggang Teng, Wei Yuan, Yuemei Zhang, Ruixin He, Fengwen Tang, Jie Pang, Bo Han, Ruijuan Chen, Yi Li

**Affiliations:** ^1^ Department of Radiation Oncology, The First Affiliated Hospital of Xi’an Jiaotong University, Xi’an, China; ^2^ Department of Radiation Oncology, Shaanxi Provincial Tumor Hospital, Affiliated Hospital of Xi’an Jiaotong University Health Science Center, Xi’an, China; ^3^ Department of Obstetrics and Gynecology, Xi’an Central Hospital, The Affiliated Hospital of Xi’an Jiaotong University, Xi’an, China; ^4^ Department of Thoracic Surgery, Second Affiliated Hospital of Air Force Medical University, Xi’an, China; ^5^ Department of Radiation Oncology, Weinan Central Hospital, Weinan, China

**Keywords:** left sided breast cancer, radiotherapy, free breathing, deep inspiration breath hold, meta-analysis

## Abstract

**Objectives:**

Modern breast cancer techniques, such as the deep inspiration breath-hold (DIBH) technique has been applied for left-sided breast cancer. Whether the DIBH regimen is the optimal solution for left-sided breast cancer remains unclear. This meta-analysis aims to elucidate the differences of DIBH and free-breathing (FB) for patients receiving radiotherapy for left-sided breast cancer and provide a practical reference for clinical practice.

**Methods:**

Relevant research available on PubMed, Embase, Cochrane Library, and the Web of Science published before November 30, 2021 was independently and systematically examined by two investigators. Data were extracted from eligible studies for assessing their qualities and calculating the standardized mean difference (SMD) and 95% confidence intervals (CIs) using Review Manager software 5.4 (RevMan 5.4).

**Results:**

Forty-one studies with a total of 3599 left-sided breast cancer patients were included in the meta-analysis. Compared with FB, DIBH reduced heart dose (*D*_mean_, *D*_max_, V30, V10, V5), left anterior descending branch (LAD) dose (*D*_mean_, *D*_max_), ipsilateral lung dose (*D*_mean_, V20, V10, V5), and heart volume significantly. Lung volume increased greatly, and a statistically significant difference. For contralateral breast mean dose, DIBH has no obvious advantage over FB. The funnel plot suggested this study has no significant publication bias.

**Conclusions:**

Although DIBH has no obvious advantage over FB in contralateral breast mean dose, it can significantly reduce heart dose, LAD dose, ipsilateral lung dose, and heart volume. Conversely, it can remarkably increase the ipsilateral lung volume. This study suggests that soon DIBH could be more widely utilized in clinical practice because of its excellent dosimetric performance.

## Introduction

Breast cancer is a significant global public health problem and the leading cause of cancer mortality in women (1). Adjuvant radiation therapy has a major role managing this disease, reducing the risk of local recurrence and breast cancer-specific mortality (2). It is certain that radiotherapy is an effective way to treat breast cancer, and significantly prolongs the survival time. However, breast cancer radiation therapy is also associated with higher cardiac and pulmonary toxicity [e.g., radiation-related heart disease (RRHD) (3) and radiation pneumonia (RP) (4)] with an increased risk of secondary cancer (3, 5–9). Darby et al. showed the risk of major coronary events induced by radiation increased linearly with the mean heart dose (MHD) by 7.4% per gray, with no threshold dose (3). Clarke et al. compared a group of irradiated patients with non-irradiated patients and found a significant increase in mortality rate, mainly for heart disease and lung cancer with a rate ratio of 1.27 and 1.78, respectively (2).

Therefore, with patients receiving radiotherapy for breast cancer substantial efforts have been made to develop techniques that reduce heart and lung dose, such as Deep inspiration breath-hold (DIBH). This simple technique reduces cardiac exposure by lung expansion which physically displaces the heart out of the treatment field. There are several approaches for performing DIBH, in particular active breath control, external infrared box marker, and optical surface monitor (10). Studies have demonstrated that DIBH, for left-sided breast cancer patients, can reduce the cardiac dose compared with free-breathing (FB) (5, 9, 11–13). It is noteworthy that the technique has high repeatability and stability in the whole treatment process (14).

Although many studies show DIBH technology is correlated to heart dose, LAD dose, ipsilateral lung dose, contralateral breast dose, heart volume, and ipsilateral lung volume, we have reached an understanding that DIBH is critical and superior to free-breathing (FB) in radiotherapy for left-sided breast cancer. However, there are many small sample studies, which gives a lack confidence. Therefore, we searched all of the controlled studies of DIBH and FB in radiotherapy of the left breast and conducted this meta-analysis. It is noteworthy that the research groups with different radiotherapy techniques (3D-CRT, IMRT, or VMAT), postures (supine or prone position), and prescribed dose schemes (CF or HF) in the same study were included in this meta-analysis.

## Methods

### Search Strategy

Using a combination of medical subject heading (MeSH) terms and/or free text words such as, “breast cancer”, “radiotherapy” and “deep inspiration breath-hold or DIBH”, we thoroughly searched four medical databases including PubMed, Embase, Cochrane library, and Web of Science for relevant studies published before November 30, 2021. There was no limitation on the language of published studies. Furthermore, references of selected studies were manually reviewed, and literature searching and screening were independently performed by two investigators. Disagreement was resolved through discussion with a third investigator.

### Inclusion Criteria

All studies included were following the principles of PICOS (Participants, Intervention, Comparison and Outcomes, Study design). Inclusion criteria were as follows: (1) Participants [P]: Patients were pathologically diagnosed with left-sided breast cancer without distant metastasis. (2) Intervention [I]: Patients in the experimental group received a DIBH regimen. (3) Comparison [C]: Free-breathing (FB) regimen was the intervention in the control group. (4) Outcomes [O]: The outcomes included dosimetric indicators of heart, left anterior descending artery, ipsilateral lung, and contralateral breast: the mean dose (*D*_mean_), the maximum dose (*D*_max_), and the percentage of the organ volume receiving at least 5 Gy (V5), 10 Gy (V10), 20 Gy (V20), 25 Gy (V25) and 30 Gy (V30). (5) Study design [S]: randomized controlled trials (RCTs) and observational studies, including cohort and case-control studies. It should be noted that trials with different fractionation regimens and prescribed doses were included in this study.

### Exclusion Criteria

Articles satisfying any of the following items were excluded: (1) Reviews, case reports, letters, and abstracts; (2) Low research quality or having a high risk of bias; (3) Lacking available data that could be pooled.

### Data Extraction

The following information was independently extracted from the included studies by two researchers (Mr. Yang and Mr. Teng): First author, year of publication, country, study design, age, DIBH type, clinical tumor stage, sample size, detailed treatment plan, and outcomes of the various subgroups. Dispute regarding data extraction was arbitrated by a third investigator (Mr. Tang).

### Quality Assessment

To assess the risk of bias in nonrandomized studies Newcastle-Ottawa Scale (NOS) (15) was introduced, involving three perspectives: Selection, comparability, and outcome of the studies. Using a 0-9 scale, 4 points were graded for selection, 2 for comparability, and 3 for outcomes. Studies with 6 points or higher were considered high quality (16).

### Statistical Analysis

The pooled statistics were performed using RevMan software version 5.4 (Cochrane Collaboration, Oxford, UK). Standardized mean difference (SMD) and 95% CI were selected as the effect indicator to analyze measurement data. Heterogeneity was evaluated between trials through the Cochrane Q test and the *I* ^2^ statistic, which quantified the proportion of total variation caused by heterogeneity instead of chance (17). If the *P*-value of the Q test was >0.10 and *I* ^2^< 50%, a fixed-effects model was used for data with non-significant heterogeneity. Otherwise, a random-effects model was used for data with significant heterogeneity (18, 19). Furthermore, the sensitivity analysis was also applied to examine the potential influence of an individual study on the overall assessment, which involved removing one study each time and pooling the remaining trials. A funnel plot was used to understand the bias of the literature publication. If the points in the funnel plot are symmetrically distributed on both sides of the middle dashed line and concentrate in the center, the possibility of publication bias is low. If not, the possibility of publication bias could be high.

## Results

### Study Selection

**Figure 1:**
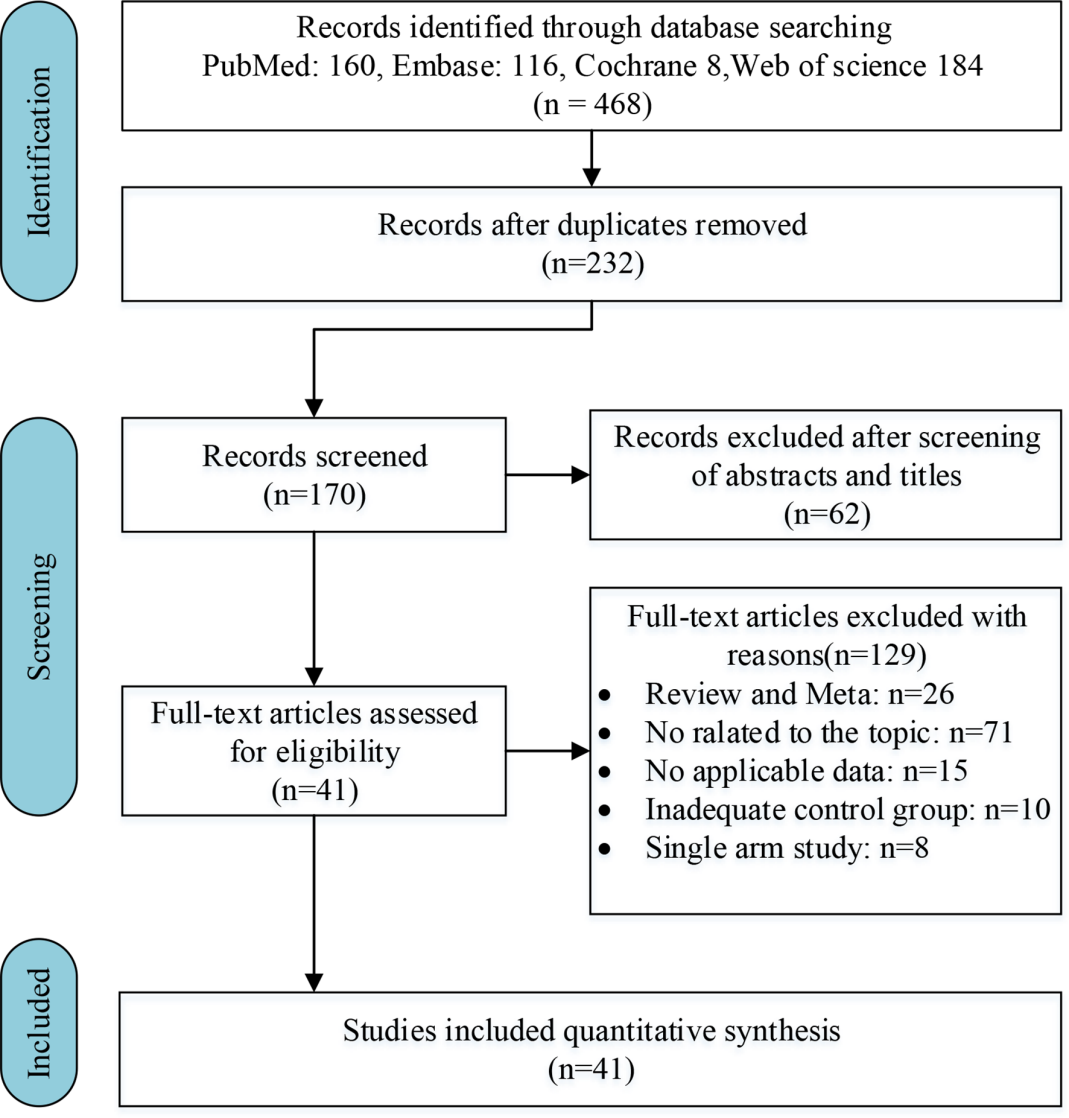
Flow chart of the search process for the meta-analysis.

Initially, after excluding 236 duplicates, 232 articles were retrieved through preliminary searches in PubMed, Embase, the Cochrane Library, and Web of Science. Then, 62 unqualified articles were eliminated through reviewing titles and abstracts. After a full-text reading, 41 qualified articles were assessed for design and quality (5, 7, 20, 13, 21–57). The detailed process of the study selection is shown in **Figure 1**.

### Study Characteristics

Finally, 41 studies (5, 7, 13, 20–57) totaling 3599 left-sided breast cancer patients were included in our meta-analysis. All articles included were retrospective studies and identified as high quality by the Newcastle–Ottawa Scale (15). **Table 1** summarizes the baselines information of the 41 included studies. Each group of data shall be counted independently when multiple groups of data are in the same study.

**Table 1:** Characteristics of the studies included in the meta-analysis.

First author (year of publication)	Total Patients (DIBH/FB)	Clinical stage	Median age (years)	Prescription dose(Gy)/Fractions(F)	DIBH types	Study type	NOS score
Angela 2017 (20)	64 (32/32)	NA	NA	50 Gy/25 F	RPM	Retrospective	6
Bruzzaniti 2013 (CF) (21)	16 (8/8)	NA	51	50 Gy/25 F	RPM	Retrospective	7
Bruzzaniti 2013 (HF) (21)	16 (8/8)	NA	51	34 Gy/10 F	RPM	Retrospective	7
Chatterjee 2018 (22)	70 (50/20)	NA	NA	40 Gy/15 F	RPM	Retrospective	6
Chi. F. 2015 (23)	62 (31/31)	I or II	39.5	50 Gy/25 F	ABC	Retrospective	8
Christina 2021 (24)	194 (97/97)	NA	54	40.05-50.4 Gy/15 -28 F	RPM	Retrospective	7
Comsa 2014 (25)	60 (30/30)	NA	<50	50 Gy/25 f	ABC	Retrospective	6
Dincoglan 2013 (26)	54 (27/27)	NA	<65	50 Gy/25 f	ABC	Retrospective	7
Dolezel 2021 (27)	200 (100/100)	cT1-3N0-2	59	48.6 Gy/27 f	NA	Retrospective	7
Eldredge 2015 (28)	172 (86/86)	T1–3N0–3M0	52	50 Gy/25 f	ABC	Retrospective	9
Ferini 2021 (29)	232 (116/116)	I-II	56	40.5-50 Gy/15-25 f	RPM	Retrospective	8
Goyal 2020 (30) (prone position)	28 (14/14)	NA	>18	40-42.6 Gy/15-16 f	RPM	Retrospective	7
Hammadi 2018 (31)	108 (54/54)	NA	41	50 Gy/25 f	NA	Retrospective	6
Hepp 2015 (32)	40 (20/20)	pTis–pT1 pN0	NA	50 Gy/25 f	Catalyst	Retrospective	7
Jensen 2017 (33)	44 (22/22)	pT1-2N0M0, ductal carcinoma	58	50 Gy/25 f	laser-based DIBH system	Retrospective	7
Jiheon 2020 (34)	150 (75/75)	Invasive breast cancer or ductal carcinoma	NA	40-42.5 Gy/15-16 f	Medspira Breath-Hold	Retrospective	7
Kunheri 2017 (35)	90 (45/45)	I–IIIA	45.2	40 Gy/15 f	ABC	Retrospective	8
Lastrucci 2017 (36)	46 (23/23)	NA	NA	50 Gy/25 f	Medspira Breath-Hold	Retrospective	7
Lawler 2017 (37)	56 (28/28)	NA	57.39	40.05–50 Gy/15–25 f	RPM	Retrospective	7
Lee 2013 (38)	50 (25/25)	≤T2 and ≤N1a	29	50.4 Gy/28f	Abches	Retrospective	8
Lin 2019 (39)	184 (63/121)	Tis, I, or II	51.53	50 Gy/25 f	ABC	Retrospective	8
Liuwei 2021 (40)	22 (11/11)	NA	NA	42.4 Gy/16f	NA	Retrospective	6
Misra 2021 (41)	60 (30/30)	I-III	50	40 Gy/15f	RPM	Retrospective	9
Mohamad 2017 (42)	44 (22/22)	NA	NA	50 Gy/25 f	ABC	Retrospective	6
Nissen 2013 (43)	227 (144/83)	NA	55.5 (DIBH) 64 (FB)	50 Gy/25 f	ABC	Retrospective	9
Pham 2016 (44) (IMRT Group)	30 (15/15)	NA	NA	50 Gy/25 f	RPM	Retrospective	6
First author (year of publication)	Total Patients (DIBH/FB)	Clinical stage	Median age (years)	Prescription dose(Gy)/Fractions(F)	DIBH types	Study type	NOS score
Pham 2016 (44) (VMAT Group)	30 (15/15)	NA	NA	50 Gy/25 f	RPM	Retrospective	6
Rochet 2015 (45)	70 (35/35)	Tis-T3N+M0	51	42.4–50-50.4 Gy/16–25-28 f	AlignRT	Retrospective	7
Saini 2018 (46)	66 (33/33)	T1-2N0	NA	42.56 Gy/16 f	DIBH (other)	Retrospective	7
Saini 2019 (7) (prone position)	50 (25/25)	T1-2N0	NA	42.56 Gy/16 f	DIBH (other)	Retrospective	7
Saini 2019 (7) (supine position)	50 (25/25)	T1-2N0	NA	42.56 Gy/16 f	DIBH (other)	Retrospective	7
Sakka 2017 (47) (IMRT Group)	40 (20/20)	NA	<70	50.4 Gy/28 f	RPM	Retrospective	7
Sakka 2017 (47) (VMAT Group)	40 (20/20)	NA	<70	50.4 Gy/28 f	RPM	Retrospective	7
Sakyanun 2020 (48)	50 (25/25)	NA	NA	50 Gy/25 f	RPM	Retrospective	6
Schönecker 2016 (49)	18 (9/9)	NA	46.9	50 Gy/25 f	Catalyst/Sentinel	Retrospective	7
Shim 2012 (50)	20 (10/10)	T1N0, T2N0,T2N1	44	50 Gy/25 f	NA	Retrospective	6
Simonetto 2019 (51)	198 (89/89)	Tis-T4	57	40-50 Gy/15-25 f	Catalyst/Sentinel	Retrospective	9
Stranzl 2009 (52)	22 (11/11)	NA	51	NA	RPM	Retrospective	6
Sunmin 2021 (53)	30 (15/15)	T1-2N0	54	50 Gy/25 f	RPM	Retrospective	9
Tanguturi 2015 (54)	148 (110/38)	All stages	58/49.5	50 Gy/25 f	AlignRT	Retrospective	8
Vikström 2011 (55)	34 (17/17)	NA	60	50 Gy/25 f	RPM	Retrospective	6
Wang 2012 (13)	106 (53/53)	NA	52	42.4–50 Gy/16–25 f	ABC	Retrospective	8
Wiant 2015 (56)	50 (25/25)	NA	NA	50.4 Gy/28 f	Philips Bellows system	Retrospective	7
Yamauchi 2020 (5)	170 (85/85)	NA	49.3	50 Gy/25 f	RPM	Retrospective	7
Zhao-Feng 2018 (57) (3D-CRT Group)	44 (22/22)	NA	48	50 Gy/25 f	RPM	Retrospective	7
Zhao-Feng 2018 (57) (IMRT Group)	44 (22/22)	NA	48	50 Gy/25 f	RPM	Retrospective	7

DIBH, deep inspiration breath hold; FB, free breathing; NOS, Newcastle–Ottawa Scale; CF, conventional fractionation; HF, Hypofractionation; ABC, active breathing coordinator; RPM, real-time position management; AlignRT, a realtime surfacetracking system; VMAT, volumetric modulated arc therapy; IMRT, intensity-modulated radiation therapy; 3D-CRT, 3-dimensional conformal radiotherapy; NA, not available.

### Heart Dose

**Figure 2:**
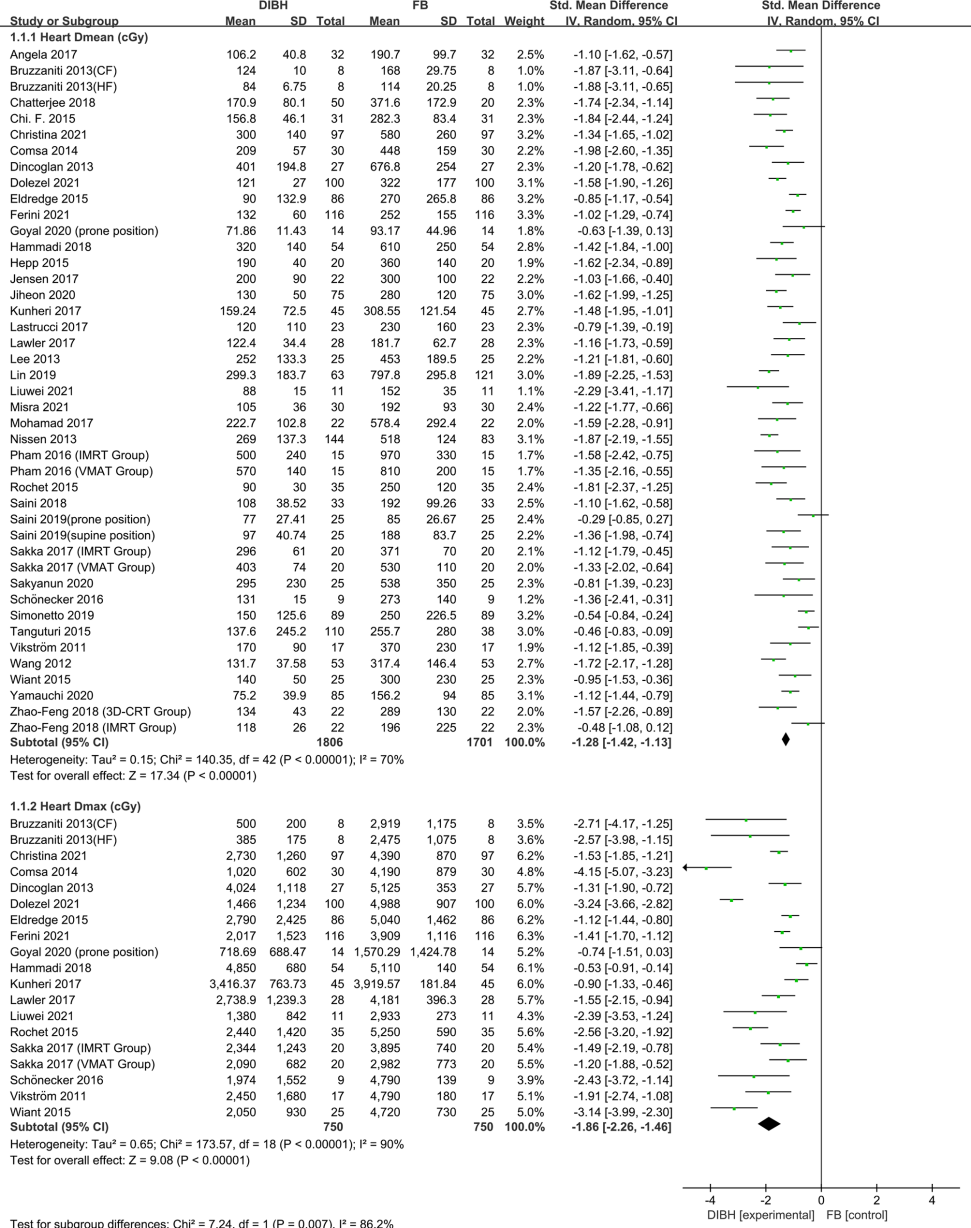
Forest plot of heart dose (*D*_mean_ and *D*max) between the DIBH group and FB group.

**Figure 3:**
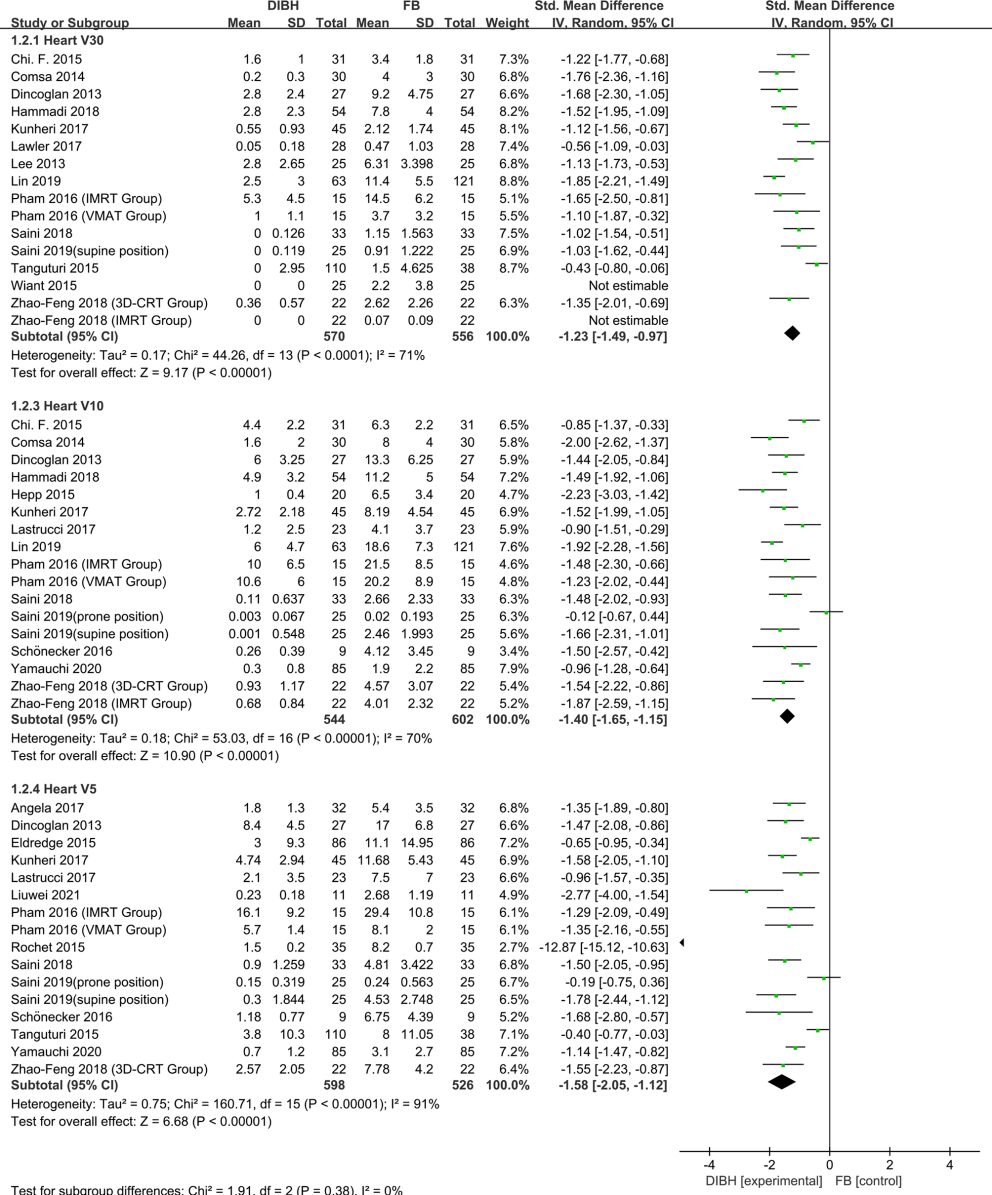
Forest plot of heart dose (V30, V10 and V5) between the DIBH group and FB group.

Heart dose data (*D*_mean_, *D*_max_, V30, V10, and V5) were extracted from 38 articles which studied 3507 patients. The random-effects model was applied due to the significant between-study heterogeneity (*I* ^2^≥50%, P ≤ 0.10). The pooled results showed there was a difference between the DIBH group and FB group. By combining the results with clinical information from the included studies, it was indicated that DIBH technology can decrease heart doses more effectively than the FB group. The results are presented in **Figures 2** and **3**, *D*_mean_ (SMD = -1.28, 95% CI: -1.42 - 1.13, P<0.01), *D*_max_ (SMD = -1.86, 95% CI: -2.26 ~ -1.46, P<0.01), V30 (SMD = -1.23, 95% CI: -1.49 ~ 0.97 P<0.01), V10 (SMD = - 1.40, 95% CI: -1.65 ~ -1.15, P<0.01), V5 (SMD = -1.58, 95% CI: -2.05 ~ -1.12, P<0.01).

### LAD Dose

**Figure 4:**
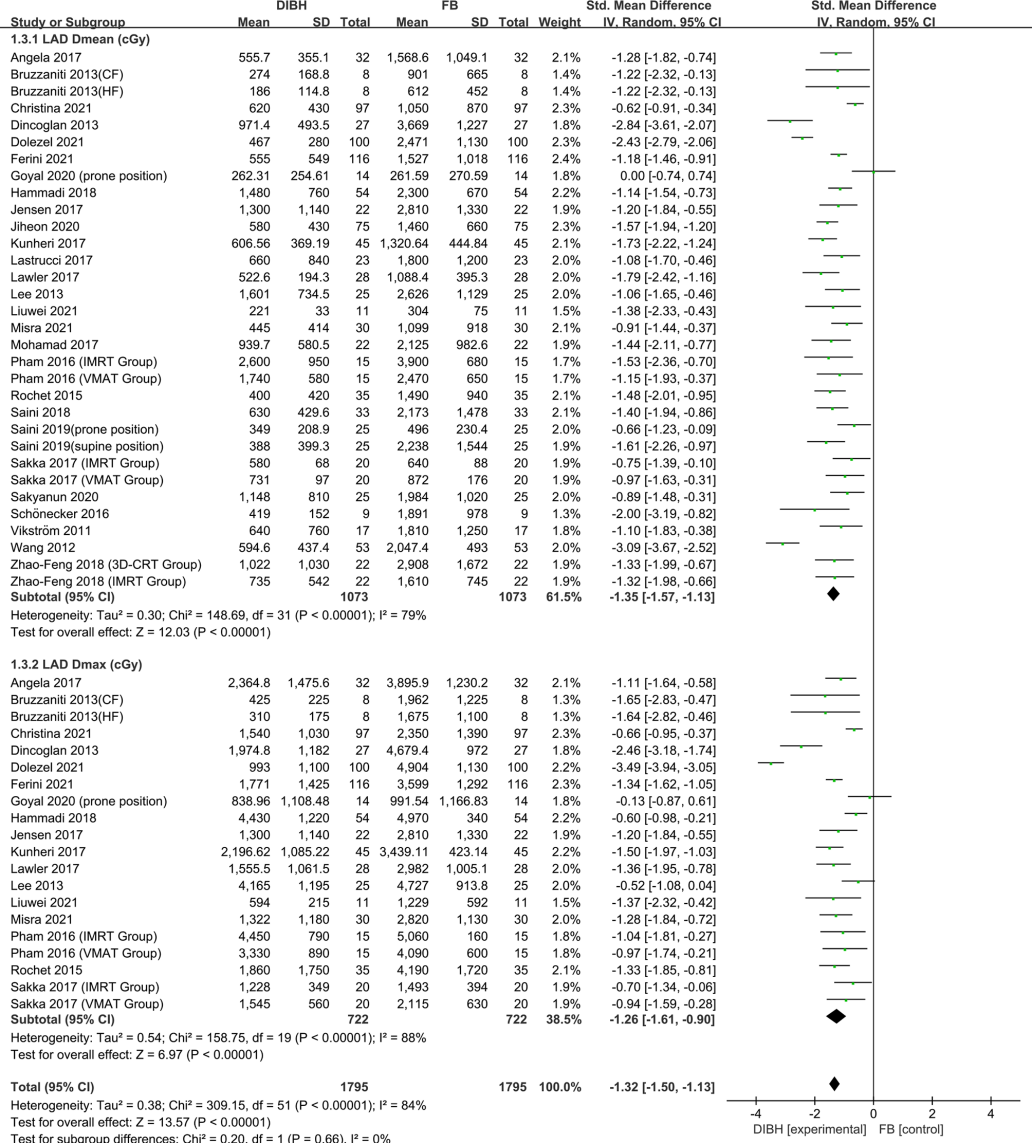
Forest plot of LAD dose (*D*_mean_ and *D*max) between the DIBH group and FB group.

Twenty-seven studies involving 2146 patients were eligible for analyzing the LAD dose (*D*_mean_ and *D*_max_). Significant heterogeneity was identified (*I* ^2^≥50%, P ≤ 0.10) and as a result, a random-effects model was employed to calculate the pooled data. The data demonstrated that the LAD dose (*D*_mean_ and *D*_max_) of the DIBH group was significantly lower than that of the FB group (*D*_mean_: SMD = -1.35, 95% CI: -1.57 ~ -1.13, P<0.01; *D*_max_: SMD = -1.26, 95% CI: -1.61 ~ -0.90, P<0.01) (**Figure 4**).

### Ipsilateral Lung Dose

**Figure 5:**
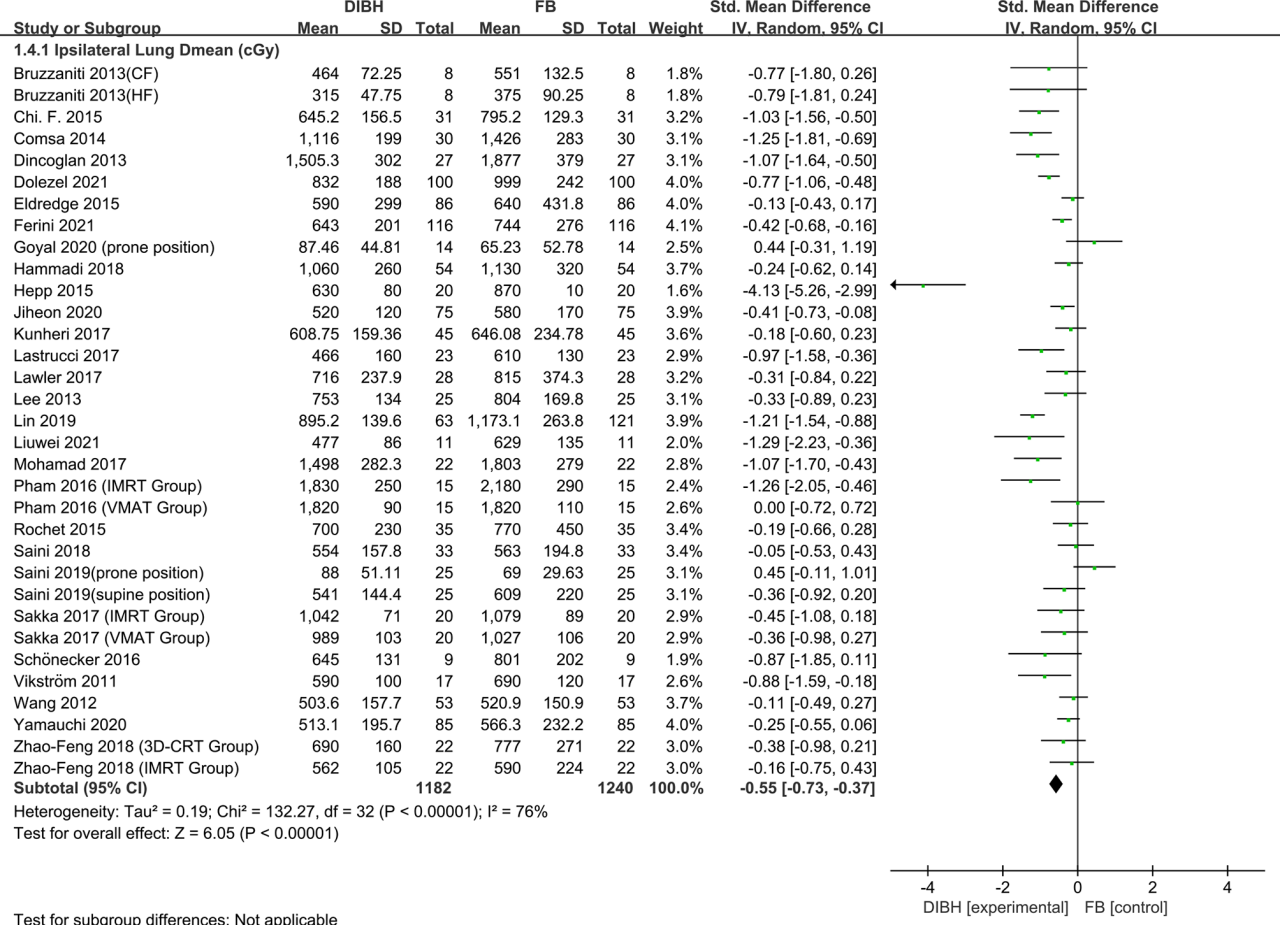
Forest plot of ipsilateral lung dose (*D*_mean_) between the DIBH group and FB group.

**Figure 6:**
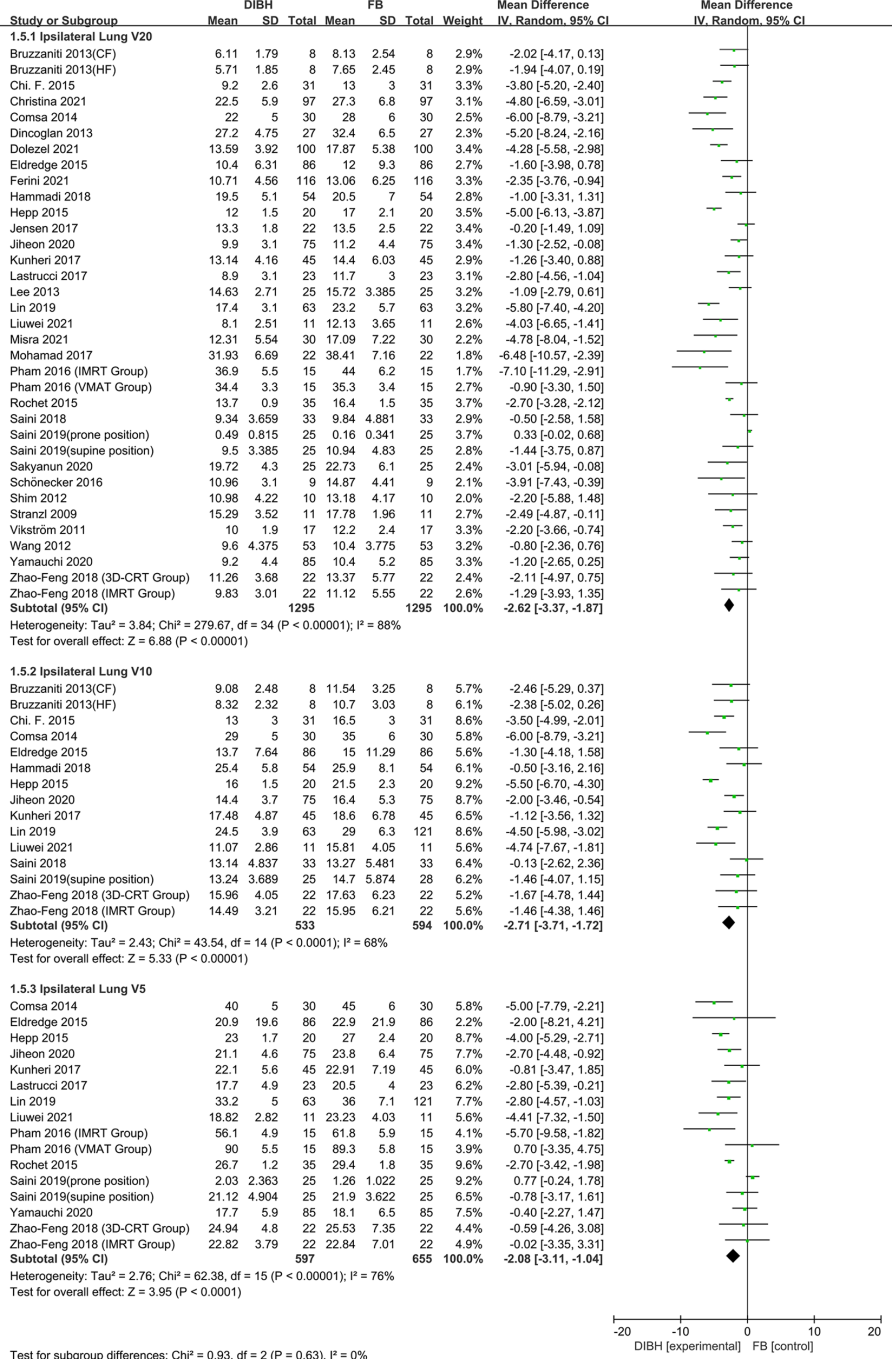
Forest plot of ipsilateral lung dose (V20, V10 and V5) between the DIBH group and FB group.

Ipsilateral lung dosimetric indicators (*D*_mean_, V20, V10, and V5) were extracted from 33 studies with 2768 patients. The heterogeneity test showed statistically significant differences among the studies (*I* ^2^ ≥ 50%, P ≤ 0.10), and therefore, a random-effects model was introduced. Compared to the FB group, left-sided breast cancer patients could benefit more from DIBH technology. The results are presented in **Figures 5** and **6**, *D*_mean_ (SMD = - 0.55, 95% CI: -0.73 ~ -0.37, P<0.01), V20 (SMD = -2.62, 95% CI: -3.37 ~ -1.87 P<0.01), V10 (SMD = -2.71, 95% CI: -3.71 ~ -1.72, P<0.01), V5 (SMD = - 2.08, 95% CI: -3.11 ~ -1.04, P<0.01).

### Contralateral Breast Mean Dose

**Figure 7:**
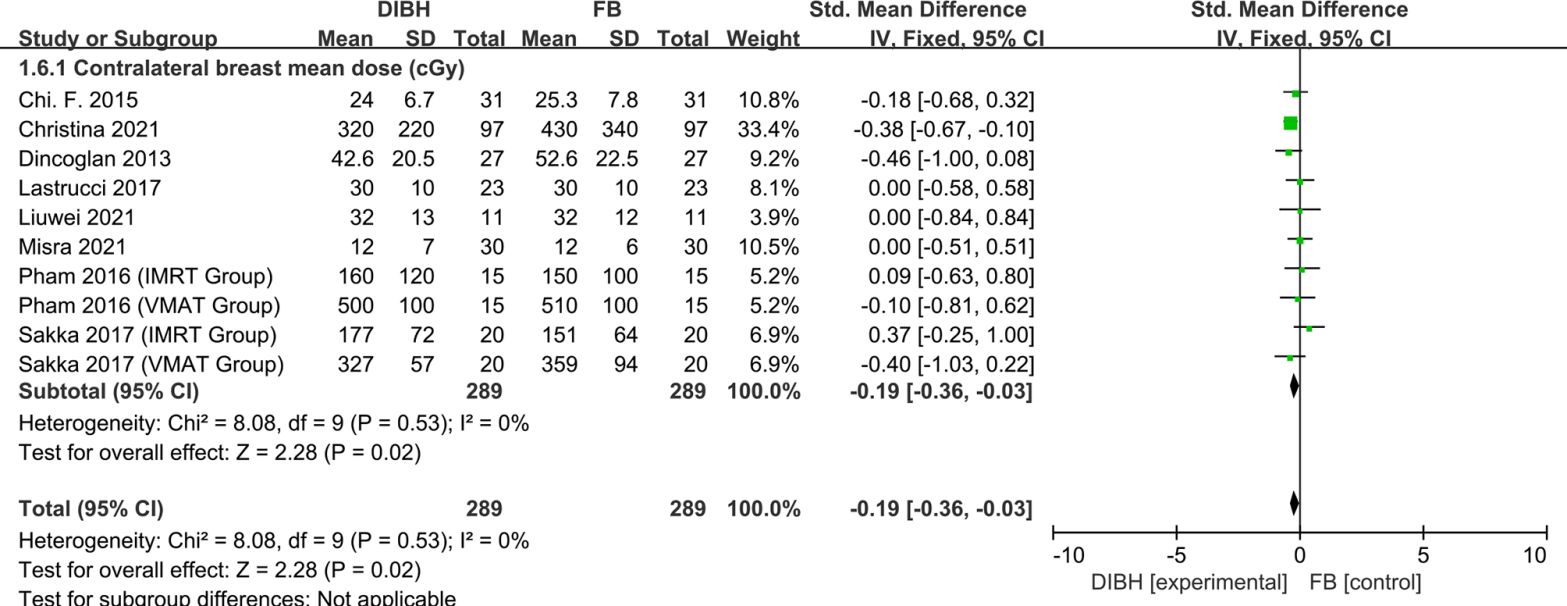
Forest plot of contralateral breast mean dose between the DIBH group and FB group.

Eight studies, with 578 left-sided breast cancer patients in total, were included in this analysis. During the analysis, we found no significant between-study heterogeneity (*I* ^2^ = 0%; p = 0.53), and a fixed-effects model was used. The combined analysis showed that there was no significant difference in contralateral breast mean dose between the two groups and there was no statistical significance (SMD = -0.19, 95% CI: -0.36 ~ -0.03, P=0.02) (**Figure 7**).

### Heart Volume

**Figure 8:**
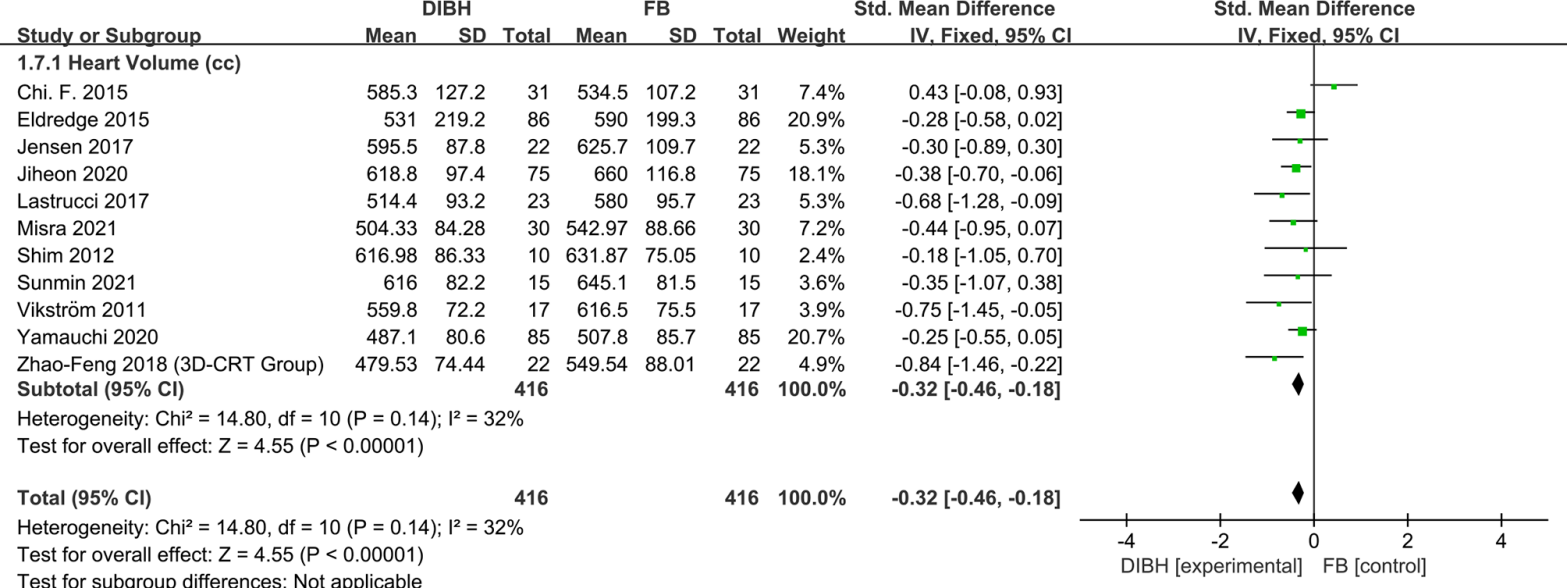
Forest plot of heart volume between the DIBH group and FB group.

Heart volume was reported in eleven studies with a total of 832 patients. The fixed-effects model was applied due to no significant between-study heterogeneity (*I* ^2^ = 32%; p = 0.14). In comparison with the FB group, the application of DIBH technology makes cardiac volume compression in patients with left-sided breast cancer. (SMD = -0.32, 95% CI: -0.46 ~ -0.18, P<0.01) (**Figure 8**).

### Ipsilateral Lung Volume

**Figure 9:**
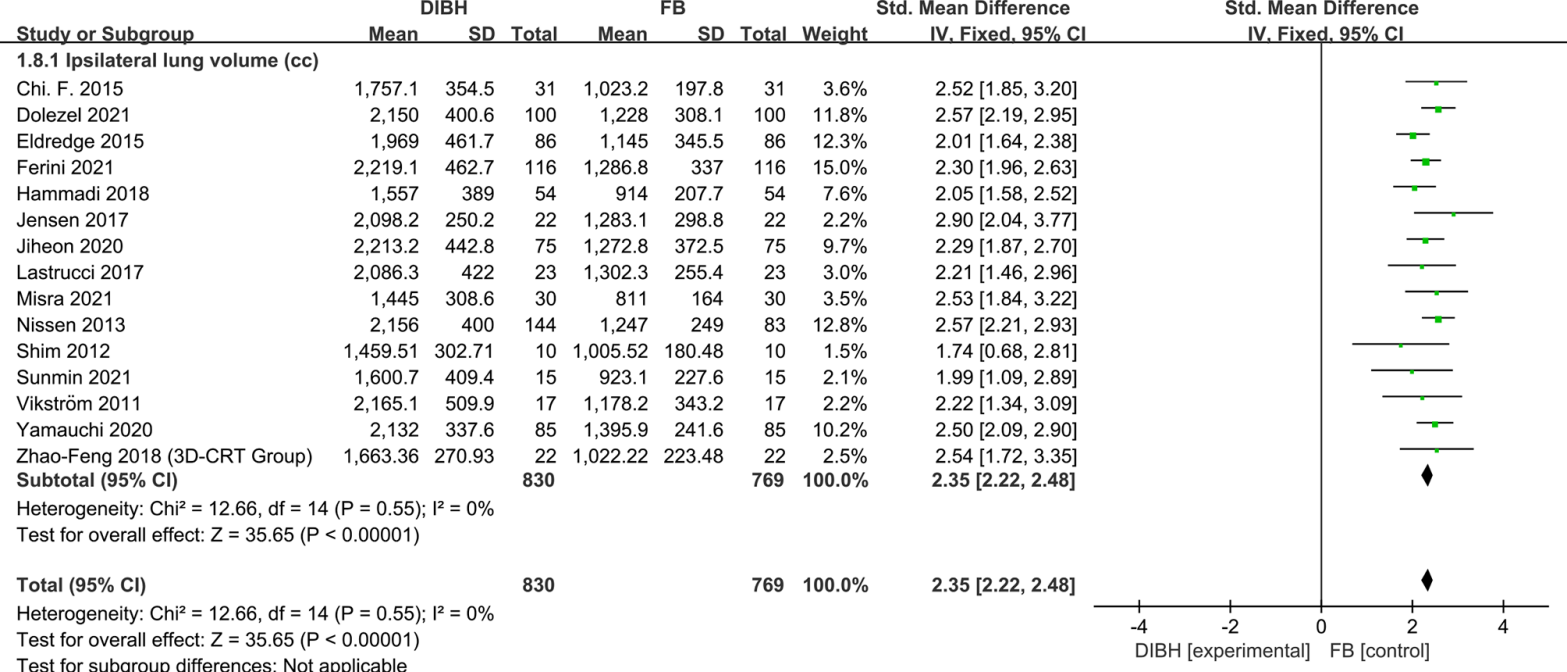
Forest plot of ipsilateral lung volume between the DIBH group and FB group.

Fifteen studies involving 1599 left-sided breast cancer patients were eligible for analysis. The fixed-effects model was conducted for no significant between-study heterogeneity (*I* ^2^ = 0%; p = 0.55). Meta-analysis showed that DIBH technology significantly increased the ipsilateral lung volume (SMD = 2.35, 95% CI: 2.22 ~ -2.48, P<0.01) (**Figure 9**).

### Publication Bias

**Figure 10:**
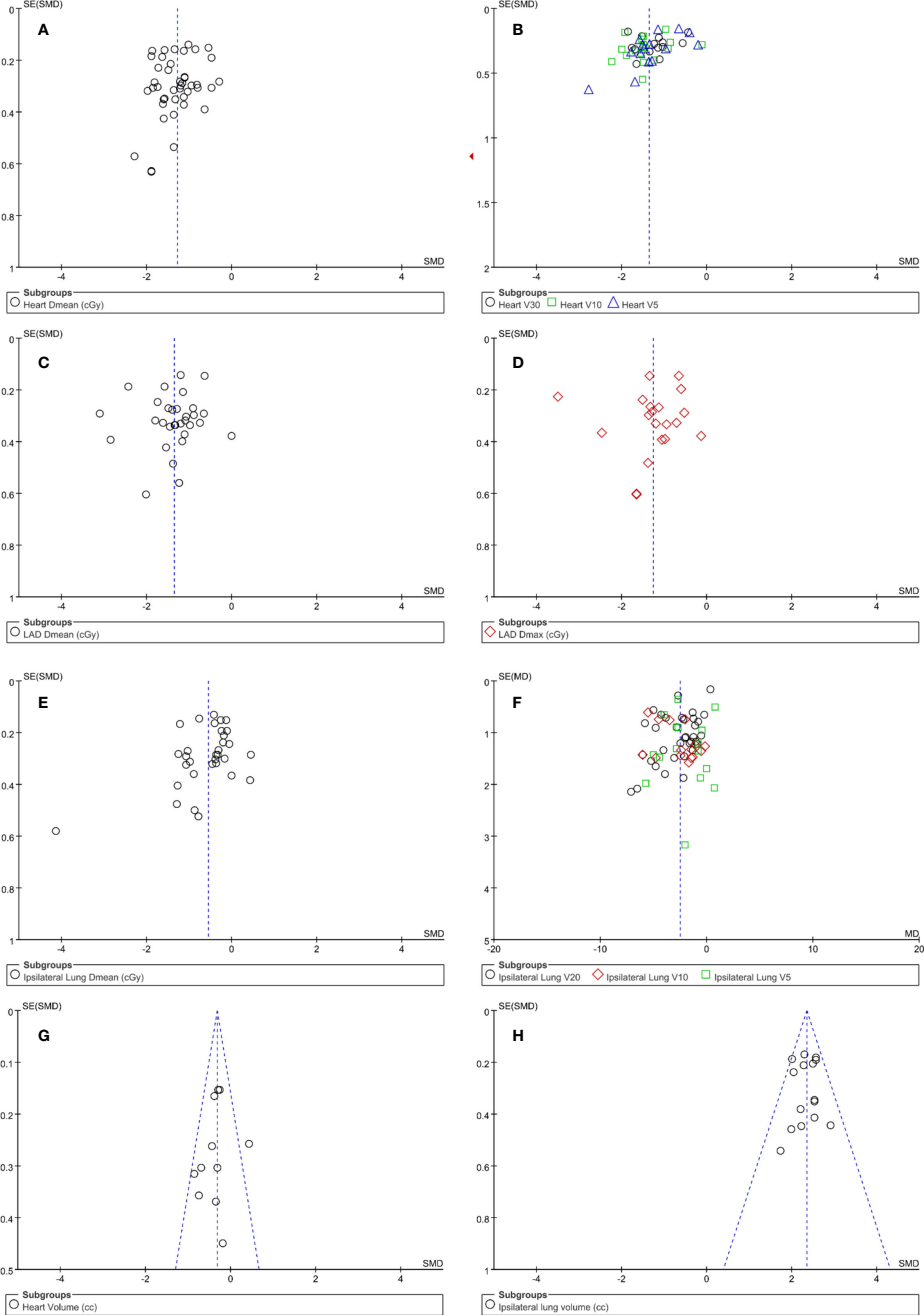
Funnel plots for potential publication bias. Funnel plot analysis of heart dose **(A, B)**, LAD dose **(C, D)**, ipsilateral lung dose **(E, F)**, heart volume **(G)** and ipsilateral lung volume **(H)**.

A funnel plot was applied for the assessment of publication bias in the literature, tests for the funnel plot asymmetry were applied if there were at least 10 studies included in the meta-analysis. From the funnel plot of different indicators (**Figure 10**), it is evident that the point estimates are symmetrically distributed on both sides, centralized in the middle, therefore showing no evidence of publication bias.

## Discussion

There are many studies on the incidence of RRHD caused by radiotherapy for breast cancer. The research of Darby et al. (3) in 2013 showed that exposure of the heart to ionizing radiation during radiotherapy for breast cancer increases the subsequent rate of ischemic heart disease. The increase is proportional to the mean dose to the heart, beginning within a few years after exposure, and continues for at least 20 years. Women with preexisting cardiac risk factors have greater absolute increases in risk from radiotherapy than other women. Additionally, further studies indicate that LAD coronary artery doses may be particularly relevant to RRHD risks, since this artery is a common site of atherosclerosis inducing myocardial infarction. It is the site of high doses in many left-breast cancer radiotherapy regimens, and may well contribute to radiation-induced heart disease (58). Some recent research has focused on the relationship between the average cardiac dose and the incidence of adverse events. One such research conducted by Van den Bogaard et al. concluded that the cumulative incidence of acute coronary events increased by 16.5% per Gy (59). A study by Dutch et al. showed that the risk of myocardial infarction increased linearly as the mean of the whole heart dose increased, with an excess risk ratio of 6.4% per Gy (60).. In another Ebbe Laugaard Lorenzen et al. study, it was demonstrated that for female patients receiving tangential field irradiation, the linear increase in the excess odds ratio of major coronary events per gray of mean heart dose was 19% (61). Therefore, to reduce the incidence of RRHD, the deposition dose of heart and LAD should be low enough. In this paper, we respectively studied the dosimetric indexes of heart and LAD. The results implied that the dose of the heart and LAD in the DIBH group was significantly lower than that in the FB group. The meta-analysis results of all subgroups of cardiac dose (*D*_mean_, *D*_max_, V30, V10, and V5) and LAD dose subgroup (*D*_mean_, *D*_max_) support this conclusion unanimously (**Figures 2**–**4**). We have reason to believe that DIBH may reduce RRHD more effectively by reducing the dose to the heart and LAD, such as ischemic heart disease, acute coronary event and myocardial infarction. Moreover, the results of this study infer that different radiotherapy techniques (3D-CRT, IMRT or VMAT), postural design (supine or prone position) and prescribed dose schemes (CF or HF) did not affect the dose reduction advantages of DIBH compared with FB in the heart and LAD.

In 1998, Kwa et al. (62) conducted a large multicenter study of 530 people on the relationship between the incidence of radiation pneumonitis and dose distribution in the lungs. Their results showed that increasing pneumonitis rate was observed with increasing mean lung dose in all centers. Especially in the low dose range of 4 to 16 Gy, the incidence rate of pneumonia in the breast group was 1.4%. Therefore, the mean lung dose can be used as a useful predictor of the risk of radiation pneumonia. Additionally, research conducted by Gokula et al. and Kasmann et al. implied that Locoregional radiotherapy increased the mean lung dose, and ipsilateral lung volume receiving 20 Gy (V20) >30% have been identified as risk factors for RP (63, 64). In this study, ipsilateral lung dosimetric indicators (*D*_mean_, V20, V10, and V5) were extracted from 33 studies totaling 2768 patients. Compared to the FB group, left-sided breast cancer patients could benefit more from DIBH technology. The subgroup analysis results are presented in **Figures 5** and **6**, *D*_mean_ (SMD = -0.55, 95%, CI: -0.73 ~ -0.37, P<0.01), V20 (SMD = -2.62, 95% CI: -3.37 ~ -1.87 P < 0.01), V10 (SMD = -2.71, 95% CI: -3.71 ~ -1.72, P <0.01), V5 (SMD = -2.08, 95% CI: -3.11 ~ -1.04, P<0.01). We can conclude that DIBH technology may reduce the incidence of RP by reducing the mean lung dose, V20, V10, and V5. However, there are a few exceptions. It can be seen from the forest plot (**Figures 5** and **6**) that DIBH did not perform better than FB in all prone position groups. Therefore, large sample size experiments are needed to focus on the difference between DIBH technology and FB in dissimilar postures. In addition, the results of this study infer that different radiotherapy techniques (3D-CRT, IMRT or VMAT) and prescribed dose schemes (CF or HF) did not affect the dose reduction advantages of DIBH compared with FB in the ipsilateral lung.

Further, we counted and analyzed the mean dose of contralateral breast, heart volume, and ipsilateral lung volume. The combined analysis showed between the two groups there was no significant difference in contralateral breast mean dose and there was no statistical significance (SMD = - 0.19, 95% CI: -0.36 ~ -0.03, P=0.02). Meanwhile, results indicated that the ipsilateral lung volume increased significantly in the DIBH group (SMD = 2.35, 95% CI: 2.22 ~ 2.48, P<0.01), while the heart volume was compressed (SMD = -0.32, 95% CI: -0.46 ~ -0.18, P<0.01). This phenomenon is not difficult to understand, because DIBH is a simple technique used to reduce cardiac exposure by lung expansion which physically displaces the heart out of the radiation field. Objectively speaking, the use of DIBH technology expands the lung volume, which in turn makes the contralateral breast farther away from the radiation field, and finally the contralateral breast should have a lower mean dose. However, in the FB group, the contralateral breast was also almost outside the field, which made the DIBH group have no significant advantage in reducing the mean breast dose compared with the FB group.

Potential limitations exist in this study, and the meta-analysis without the distinction of surgical operation is an obvious one. In left-sided breast cancer patients with modified radical mastectomy (MRM), the target (i.e., chest wall) lies near the heart and LAD, as compared to those patients undergoing breast conservation surgery (BCS). Recently, a small sample study by Misra et al. showed that DIBH provided a similar percentage reduction in cardiopulmonary doses for both MRM and BCS. Significant reductions in mean heart dose were seen in both groups. Although lung and LAD doses were significantly reduced in MRM, lung dosimetric constraints were less frequently achieved in the subgroup with nodal radiation. Given that, we appeal to researchers to conduct more studies about the relationship between surgical methods and the benefits of DIBH technology, enabling more left-sided breast cancer patients to benefit from the development of precision medicine.

Apart from the distinction of surgical operation mode, other potential limitations are still prevalent in this study: (1) The data from the included studies were from the published articles instead of the original information of the individual patient; (2) all included articles are the retrospective studies, and the evidence level is lower than that of prospective randomized clinical trials; (3) the number of included studies is relatively small, especially for contralateral breast mean dose, which may cause bias results; (4) the heterogeneity of aggregated results were significant, and the random-effects model was applied to most indicators.

## Conclusions

In summary, this study provides a large-scale and comprehensive meta-analysis between the dosimetric parameters of DIBH and FB for left-sided breast cancer. Although DIBH has no obvious advantage over FB in contralateral breast mean dose, it can significantly reduce the heart dose, LAD dose, ipsilateral lung dose, heart volume, and substantially increase the ipsilateral lung volume. This study suggests that DIBH may be more widely used in clinical practice soon because of its excellent dosimetric performance.

## Data Availability Statement

The original contributions presented in the study are included in the article/supplementary material. Further inquiries can be directed to the corresponding author.

## Author Contributions

YKL, DY, and XZ: conceptualization. YL, WY, YZ, FT: data curation and original draft writing. YKL, YT, RH: statistical analysis. YL, JP, BH, RC: manuscript review and editing. All authors contributed to the article and approved the submitted version.

## Conflict of Interest

The authors declare that the research was conducted in the absence of any commercial or financial relationships that could be construed as a potential conflict of interest.

## Publisher’s Note

All claims expressed in this article are solely those of the authors and do not necessarily represent those of their affiliated organizations, or those of the publisher, the editors and the reviewers. Any product that may be evaluated in this article, or claim that may be made by its manufacturer, is not guaranteed or endorsed by the publisher.

## References

[1] Siegel RL Miller KD Fuchs HE Jemal A. Cancer Statistics, 2021. CA Cancer J Clin (2021) 71(1):7–33. doi: 10.3322/caac.21654 33433946

[2] Clarke M Collins R Darby S Davies C Elphinstone P Evans V. Effects of Radiotherapy and of Differences in the Extent of Surgery for Early Breast Cancer on Local Recurrence and 15-Year Survival: An Overview of the Randomised Trials. Lancet (2005) 366(9503):2087–106. doi: 10.1016/s0140-6736(05)67887-7 16360786

[3] Darby SC Ewertz M McGale P Bennet AM Blom-Goldman U Brønnum D. Risk of Ischemic Heart Disease in Women After Radiotherapy for Breast Cancer. N Engl J Med (2013) 368(11):987–98. doi: 10.1056/NEJMoa1209825 23484825

[4] Cox JD Stetz J Pajak TF. Toxicity Criteria of the Radiation Therapy Oncology Group (RTOG) and the European Organization for Research and Treatment of Cancer (EORTC). Int J Radiat Oncol Biol Phys (1995) 31(5):1341–6. doi: 10.1016/0360-3016(95)00060-c 7713792

[5] Yamauchi R Mizuno N Itazawa T Saitoh H Kawamori J. Dosimetric Evaluation of Deep Inspiration Breath Hold for Left-Sided Breast Cancer: Analysis of Patient-Specific Parameters Related to Heart Dose Reduction. J Radiat Res (2020) 61(3):447–56. doi: 10.1093/jrr/rraa006 PMC7299269 32100831

[6] Rutqvist LE Lax I Fornander T Johansson H. Cardiovascular Mortality In A Randomized Trial Of Adjuvant Radiation-Therapy Versus Surgery Alone In Primary Breast-Cancer. Int J Radiat Oncol Biol Phys (1992) 22(5):887–96. doi: 10.1016/0360-3016(92)90784-f 1555981

[7] Saini AS Das IJ Hwang CS Biagioli MC Lee WE 3rd. Biological Indices Evaluation of Various Treatment Techniques for Left-Sided Breast Treatment. Pract Radiat Oncol (2019) 9(6):e579–90. doi: 10.1016/j.prro.2019.06.020 31283990

[8] Grantzau T Thomsen MS Væth M Overgaard J. Risk of Second Primary Lung Cancer in Women After Radiotherapy for Breast Cancer. Radiother Oncol (2014) 111(3):366–73. doi: 10.1016/j.radonc.2014.05.004 24909095

[9] Hayden AJ Rains M Tiver K. Deep Inspiration Breath Hold Technique Reduces Heart Dose From Radiotherapy for Left-Sided Breast Cancer. J Med Imaging Radiat Oncol (2012) 56(4):464–72. doi: 10.1111/j.1754-9485.2012.02405.x 22883657

[10] Shah C Badiyan S Berry S Khan AJ Goyal S Schulte K. Cardiac Dose Sparing and Avoidance Techniques in Breast Cancer Radiotherapy. Radiother Oncol (2014) 112(1):9–16. doi: 10.1016/j.radonc.2014.04.009 24813095

[11] Yeung R Conroy L Long K Walrath D Li H Smith W. Cardiac Dose Reduction With Deep Inspiration Breath Hold for Left-Sided Breast Cancer Radiotherapy Patients With and Without Regional Nodal Irradiation. Radiat Oncol (2015) 10(1):200. doi: 10.1186/s13014-015-0511-8 26391237 PMC4578779

[12] McIntosh A Shoushtari AN Benedict SH Read PW Wijesooriya K. Quantifying the Reproducibility of Heart Position During Treatment and Corresponding Delivered Heart Dose in Voluntary Deep Inhalation Breath Hold for Left Breast Cancer Patients Treated With External Beam Radiotherapy. Int J Radiat OncolBiolPhys (2011) 81(4):e569–76. doi: 10.1016/j.ijrobp.2011.01.044 21531087

[13] Wang W Purdie TG Rahman M Marshall A Liu F-F Fyles A. Rapid Automated Treatment Planning Process to Select Breast Cancer Patients for Active Breathing Control to Achieve Cardiac Dose Reduction. Int J Radiat OncolBiolPhys (2012) 82(1):386–93. doi: 10.1016/j.ijrobp.2010.09.026 21093165

[14] Bartlett FR Colgan RM Carr K Donovan EM McNair HA Locke I. The UK HeartSpare Study: Randomised Evaluation of Voluntary Deep-Inspiratory Breath-Hold in Women Undergoing Breast Radiotherapy. Radiother Oncol (2013) 108(2):242–7. doi: 10.1016/j.radonc.2013.04.021 23726115

[15] Shuster J. Review: Cochrane Handbook for Systematic Reviews for Interventions, Version 5.1.0, Published 3/2011. Julian P.T. Higgins and Sally Green, Editors. Res Synthesis Methods (2011) 2(2):126–30. doi: 10.1002/jrsm.38

[16] Stang A. Critical Evaluation of the Newcastle-Ottawa Scale for the Assessment of the Quality of Nonrandomized Studies in Meta-Analyses. Eur J Epidemiol (2010) 25(9):603–5. doi: 10.1007/s10654-010-9491-z 20652370

[17] Higgins JPT Thomas J Chandler J Cumpston M Li T Page MJ Welch VA. Cochrane Handbook for Systematic Reviews of Interventions Version 6.1 (Updated September 2020). In: Cochrane (2020). Available at: www.training.cochrane.org/handbook.

[18] DerSimonian R Laird N. Meta-Analysis in Clinical Trials. Controlled Clin Trials (1986) 7(3):177–88. doi: 10.1016/0197-2456(86)90046-2 3802833

[19] Mantel N Haenszel W. Statistical Aspects of the Analysis of Data From Retrospective Studies of Disease. J Natl Cancer Inst (1959) 22(4):719–48. doi: 10.1016/0021-9681(79)90031-6 13655060

[20] Lin A Sharieff W Juhasz J Whelan T Kim DH. The Benefit of Deep Inspiration Breath Hold: Evaluating Cardiac Radiation Exposure in Patients After Mastectomy and After Breast-Conserving Surgery. Breast Cancer (Tokyo Japan) (2017) 24(1):86–91. doi: 10.1007/s12282-016-0676-5 26886584

[21] Bruzzaniti V Abate A Pinnarò P D’Andrea M Infusino E Landoni V. Dosimetric and Clinical Advantages of Deep Inspiration Breath-Hold (DIBH) During Radiotherapy of Breast Cancer. J Exp Clin Cancer Res: CR (2013) 32(1):88. doi: 10.1186/1756-9966-32-88 24423396 PMC3826503

[22] Chatterjee S Chakraborty S Moses A Nallathambi C Mahata A Mandal S. Resource Requirements and Reduction in Cardiac Mortality From Deep Inspiration Breath Hold (DIBH) Radiation Therapy for Left Sided Breast Cancer Patients: A Prospective Service Development Analysis. Pract Radiat Oncol (2018) 8(6):382–7. doi: 10.1016/j.prro.2018.03.007 29699893

[23] Chi F Wu S Zhou J Li F Sun J Lin Q. Dosimetric Comparison of Moderate Deep Inspiration Breath-Hold and Free-Breathing Intensity-Modulated Radiotherapy for Left-Sided Breast Cancer. Cancer Radiotherapie: J la Societe Francaise Radiotherapie Oncologique (2015) 19(3):180–6. doi: 10.1016/j.canrad.2015.01.003 25921618

[24] Schröder C Kirschke S Blank E Rohrberg S Förster R Buchali A. Deep Inspiration Breath-Hold for Patients With Left-Sided Breast Cancer – A One-Fits-All Approach? A Prospective Analysis of Patient Selection Using Dosimetrical and Practical Aspects. Br J Radiol (2021) 94:20210295. doi: 10.1259/bjr.20210295 PMC10996328 34111954

[25] Comsa D Barnett E Le K Mohamoud G Zaremski D Fenkell L. Introduction of Moderate Deep Inspiration Breath Hold for Radiation Therapy of Left Breast: Initial Experience of a Regional Cancer Center. Pract Radiat Oncol (2014) 4(5):298–305. doi: 10.1016/j.prro.2013.10.006 25194098

[26] Dincoglan F Beyzadeoglu M Sager O Oysul K Kahya YE Gamsiz H. Dosimetric Evaluation of Critical Organs at Risk in Mastectomized Left-Sided Breast Cancer Radiotherapy Using Breath-Hold Technique. Tumori (2013) 99(1):76–82. doi: 10.1700/1248.13792 23549004

[27] Dolezel M Odrazka K Vanasek J. Deep-Inspiration Breath Hold Radiotherapy in Patients With Left-Sided Breast Cancer After Partial Mastectomy. Rozhledy v Chirurgii: Mesicnik Ceskoslovenske Chirurgicke Spolecnosti (2021) 100(4):180–5. doi: 10.33699/pis.2021.100.4 34182760

[28] Eldredge-Hindy H Lockamy V Crawford A Nettleton V Werner-Wasik M Siglin J. Active Breathing Coordinator Reduces Radiation Dose to the Heart and Preserves Local Control in Patients With Left Breast Cancer: Report of a Prospective Trial. Pract Radiat Oncol (2015) 5(1):4–10. doi: 10.1016/j.prro.2014.06.004 25567159 PMC4289538

[29] Ferini G Molino L Tripoli A Valenti V Illari SI Marchese VA. Anatomical Predictors of Dosimetric Advantages for Deep-Inspiration-Breath-Hold 3D-Conformal Radiotherapy Among Women With Left Breast Cancer. Anticancer Res (2021) 41(3):1529–38. doi: 10.21873/anticanres.14912 33788746

[30] Goyal U Saboda K Roe D Gonzalez VJ. Prone Positioning With Deep Inspiration Breath Hold for Left Breast Radiotherapy. Clin Breast Cancer (2020) 21(4):e295–301. doi: 10.1016/j.clbc.2020.11.004 33358601

[31] Al-Hammadi N Caparrotti P Naim C Hayes J Rebecca Benson K Vasic A. Voluntary Deep Inspiration Breath-Hold Reduces the Heart Dose Without Compromising the Target Volume Coverage During Radiotherapy for Left-Sided Breast Cancer. Radiol Oncol (2018) 52(1):112–20. doi: 10.1515/raon-2018-0008 PMC5839089 29520213

[32] Hepp R Ammerpohl M Morgenstern C Nielinger L Erichsen P Abdallah A. Deep Inspiration Breath-Hold (DIBH) Radiotherapy in Left-Sided Breast Cancer: Dosimetrical Comparison and Clinical Feasibility in 20 Patients. Strahlentherapie und Onkologie: Organ der Deutschen Rontgengesellschaft [et al] (2015) 191(9):710–6. doi: 10.1007/s00066-015-0838-y 25893323

[33] Jensen CA Abramova T Frengen J Lund J. Monitoring Deep Inspiration Breath Hold for Left-Sided Localized Breast Cancer Radiotherapy With an in-House Developed Laser Distance Meter System. J Appl Clin Med Phys (2017) 18(5):117–23. doi: 10.1002/acm2.12137 PMC5875834 28755403

[34] Song J Tang T Caudrelier JM Bélec J Chan J Lacasse P. Dose-Sparing Effect of Deep Inspiration Breath Hold Technique on Coronary Artery and Left Ventricle Segments in Treatment of Breast Cancer. Radiother Oncol: J Eur Soc Ther Radiol Oncol (2020) 154:101–9. doi: 10.1016/j.radonc.2020.09.019 32950530

[35] Kunheri B Kotne S Nair SS Makuny D. A Dosimetric Analysis of Cardiac Dose With or Without Active Breath Coordinator Moderate Deep Inspiratory Breath Hold in Left Sided Breast Cancer Radiotherapy. J Cancer Res Ther (2017) 13(1):56–61. doi: 10.4103/jcrt.JCRT_1414_16 28508834

[36] Lastrucci L Borghesi S Bertocci S Gasperi C Rampini A Buonfrate G. Advantage of Deep Inspiration Breath Hold in Left-Sided Breast Cancer Patients Treated With 3D Conformal Radiotherapy. Tumori (2017) 103(1):72–5. doi: 10.5301/tj.5000563 27716875

[37] Lawler G Leech M. Dose Sparing Potential of Deep Inspiration Breath-Hold Technique for Left Breast Cancer Radiotherapy Organs-At-Risk. Anticancer Res (2017) 37(2):883–90. doi: 10.21873/anticanres.11394 28179347

[38] Lee HY Chang JS Lee IJ Park K Kim YB Suh CO. The Deep Inspiration Breath Hold Technique Using Abches Reduces Cardiac Dose in Patients Undergoing Left-Sided Breast Irradiation. Radiat Oncol J (2013) 31(4):239–46. doi: 10.3857/roj.2013.31.4.239 PMC3912239 24501713

[39] Lin CH Lin LC Que J Ho CH. A Seven-Year Experience of Using Moderate Deep Inspiration Breath-Hold for Patients With Early-Stage Breast Cancer and Dosimetric Comparison. Medicine (2019) 98(19):e15510. doi: 10.1097/md.0000000000015510 31083193 PMC6531160

[40] Tang L Ishikawa Y Ito K Yamamoto T Umezawa R Jingu K. Evaluation of DIBH and VMAT in Hypofractionated Radiotherapy for Left-Sided Breast Cancers After Breast-Conserving Surgery: A Planning Study. Technol Cancer Res Treat (2021) 20:15330338211048706. doi: 10.1177/15330338211048706 34657495 PMC8521420

[41] Misra S Mishra A Lal P Srivastava R Verma M Senthil Kumar SK. Cardiac Dose Reduction Using Deep Inspiratory Breath Hold (DIBH) in Radiation Treatment of Left Sided Breast Cancer Patients With Breast Conservation Surgery and Modified Radical Mastectomy. J Med Imaging Radiat Sci (2021) 52(1):57–67. doi: 10.1016/j.jmir.2020.12.004 33509700

[42] Mohamad O Shiao J Zhao B Roach K Ramirez E Vo DT. Deep Inspiration Breathhold for Left-Sided Breast Cancer Patients With Unfavorable Cardiac Anatomy Requiring Internal Mammary Nodal Irradiation. Pract Radiat Oncol (2017) 7(6):e361–7. doi: 10.1016/j.prro.2017.04.006 28666899

[43] Nissen HD Appelt AL. Improved Heart, Lung and Target Dose With Deep Inspiration Breath Hold in a Large Clinical Series of Breast Cancer Patients. Radiother Oncol: J Eur Soc Ther Radiol Oncol (2013) 106(1):28–32. doi: 10.1016/j.radonc.2012.10.016 23199652

[44] Pham TT Ward R Latty D Owen C Gebski V Chojnowski J. Left-Sided Breast Cancer Loco-Regional Radiotherapy With Deep Inspiration Breath-Hold: Does Volumetric-Modulated Arc Radiotherapy Reduce Heart Dose Further Compared With Tangential Intensity-Modulated Radiotherapy? J Med Imaging Radiat Oncol (2016) 60(4):545–53. doi: 10.1111/1754-9485.12459 27094588

[45] Rochet N Drake JI Harrington K Wolfgang JA Napolitano B Sadek BT. Deep Inspiration Breath-Hold Technique in Left-Sided Breast Cancer Radiation Therapy: Evaluating Cardiac Contact Distance as a Predictor of Cardiac Exposure for Patient Selection. Pract Radiat Oncol (2015) 5(3):e127–e34. doi: 10.1016/j.prro.2014.08.003 25413399

[46] Saini AS Hwang CS Biagioli MC Das IJ. Evaluation of Sparing Organs at Risk (OARs) in Left-Breast Irradiation in the Supine and Prone Positions and With Deep Inspiration Breath-Hold. J Appl Clin Med Phys (2018) 19(4):195–204. doi: 10.1002/acm2.12382 29927027 PMC6036360

[47] Sakka M Kunzelmann L Metzger M Grabenbauer GG. Cardiac Dose-Sparing Effects of Deep-Inspiration Breath-Hold in Left Breast Irradiation: Is IMRT More Beneficial Than VMAT? Strahlentherapie und Onkologie: Organ der Deutschen Rontgengesellschaft [et al] (2017) 193(10):800–11. doi: 10.1007/s00066-017-1167-0 28646251

[48] Sakyanun P Saksornchai K Nantavithya C Chakkabat C Shotelersuk K. The Effect of Deep Inspiration Breath-Hold Technique on Left Anterior Descending Coronary Artery and Heart Dose in Left Breast Irradiation. Radiat Oncol J (2020) 38(3):181–8. doi: 10.3857/roj.2020.00094 PMC7533398 33012146

[49] Schönecker S Walter F Freislederer P Marisch C Scheithauer H Harbeck N. Treatment Planning and Evaluation of Gated Radiotherapy in Left-Sided Breast Cancer Patients Using the Catalyst(TM)/Sentinel(TM) System for Deep Inspiration Breath-Hold (DIBH). Radiat Oncol (London England) (2016) 11(1):143. doi: 10.1186/s13014-016-0716-5 PMC5080745 27784326

[50] Shim JG Kim JK Park W Seo JM Hong CS Song KW. Dose-Volume Analysis of Lung and Heart According to Respiration in Breast Cancer Patients Treated With Breast Conserving Surgery. J Breast Cancer (2012) 15(1):105–10. doi: 10.4048/jbc.2012.15.1.105 PMC3318161 22493636

[51] Simonetto C Eidemüller M Gaasch A Pazos M Schönecker S Reitz D. Does Deep Inspiration Breath-Hold Prolong Life? Individual Risk Estimates of Ischaemic Heart Disease After Breast Cancer Radiotherapy. Radiother Oncol: J Eur Soc Ther Radiol Oncol (2019) 131:202–7. doi: 10.1016/j.radonc.2018.07.024 30097250

[52] Stranzl H Zurl B Langsenlehner T Kapp KS. Wide Tangential Fields Including the Internal Mammary Lymph Nodes in Patients With Left-Sided Breast Cancer. Influence of Respiratory-Controlled Radiotherapy (4D-CT) on Cardiac Exposure. Strahlentherapie und Onkologie: Organ der Deutschen Rontgengesellschaft [et al] (2009) 185(3):155–60. doi: 10.1007/s00066-009-1939-2 19330291

[53] Park S Rim CH Yoon WS. Variation of Heart and Lung Radiation Doses According to Setup Uncertainty in Left Breast Cancer. Radiat Oncol (2021) 16(1):78. doi: 10.1186/s13014-021-01806-5 33879201 PMC8056628

[54] Tanguturi SK Lyatskaya Y Chen Y Catalano PJ Chen MH Yeo WP. Prospective Assessment of Deep Inspiration Breath-Hold Using 3-Dimensional Surface Tracking for Irradiation of Left-Sided Breast Cancer. Pract Radiat Oncol (2015) 5(6):358–65. doi: 10.1016/j.prro.2015.06.002 26231594

[55] Vikström J Hjelstuen MH Mjaaland I Dybvik KI. Cardiac and Pulmonary Dose Reduction for Tangentially Irradiated Breast Cancer, Utilizing Deep Inspiration Breath-Hold With Audio-Visual Guidance, Without Compromising Target Coverage. Acta Oncol (Stockholm Sweden) (2011) 50(1):42–50. doi: 10.3109/0284186x.2010.512923 20843181

[56] Wiant D Wentworth S Liu H Sintay B. How Important Is a Reproducible Breath Hold for Deep Inspiration Breath Hold Breast Radiation Therapy? Int J Radiat Oncol Biol Phys (2015) 93(4):901–7. doi: 10.1016/j.ijrobp.2015.06.010 26530760

[57] Zhao F Shen J Lu Z Luo Y Yao G Bu L. Abdominal DIBH Reduces the Cardiac Dose Even Further: A Prospective Analysis. Radiat Oncol (London England) (2018) 13(1):116. doi: 10.1186/s13014-018-1062-6 PMC6013896 29929560

[58] Taylor CW Nisbet A McGale P Goldman U Darby SC Hall P. Cardiac Doses From Swedish Breast Cancer Radiotherapy Since the 1950s. Radiother Oncol: J Eur Soc Ther Radiol Oncol (2009) 90(1):127–35. doi: 10.1016/j.radonc.2008.09.029 19008005

[59] van den Bogaard VAB Ta BDP van der Schaaf A Bouma AB Middag AMH Bantema-Joppe EJ. Validation and Modification of a Prediction Model for Acute Cardiac Events in Patients With Breast Cancer Treated With Radiotherapy Based on Three-Dimensional Dose Distributions to Cardiac Substructures. J Clin Oncol (2017) 35(11):1171–8. doi: 10.1200/JCO.2016.69.8480 PMC5455600 28095159

[60] Jacobse JN Duane FK Boekel NB Schaapveld M Hauptmann M Hooning MJ. Radiation Dose-Response for Risk of Myocardial Infarction in Breast Cancer Survivors. Int J Radiat Oncol Biol Phys (2019) 103(3):595–604. doi: 10.1016/j.ijrobp.2018.10.025 30385276 PMC6361769

[61] Laugaard Lorenzen E Christian Rehammar J Jensen M-B Ewertz M Brink C. Radiation-Induced Risk of Ischemic Heart Disease Following Breast Cancer Radiotherapy in Denmark, 1977–2005. Radiother Oncol (2020) 152:103–10. doi: 10.1016/j.radonc.2020.08.007 32858067

[62] Kwa SLS Lebesque JV Theuws JCM Marks LB Munley MT Bentel G. Radiation Pneumonitis as a Function of Mean Lung Dose: An Analysis of Pooled Data of 540 Patients. Int J Radiat Oncol Biol Phys (1998) 42(1):1–9. doi: 10.1016/S0360-3016(98)00196-5 9747813

[63] Gokula K Earnest A Wong LC. Meta-Analysis of Incidence of Early Lung Toxicity in 3-Dimensional Conformal Irradiation of Breast Carcinomas. Radiat Oncol (2013) 8 1–12. doi: 10.1186/1748-717x-8-268 24229418 PMC3842634

[64] Kasmann L Dietrich A Staab-Weijnitz CA Manapov F Behr J Rimner A. Radiation-Induced Lung Toxicity - Cellular and Molecular Mechanisms of Pathogenesis, Management, and Literature Review. Radiat Oncol (2020) 15(1):1–16. doi: 10.1186/s13014-020-01654-9 PMC7488099 32912295

